# Hydrogen sulfide against preeclampsia exposure-induced oxidative mitochondrial damage in HTR-8/SVneo cells

**DOI:** 10.3389/fcvm.2022.1023982

**Published:** 2022-10-26

**Authors:** Xianli Wang, Shaokun Yu, Yuting Jian, Hongmin Pan, Jiannan Guo, Jian Wu, Wei Guo

**Affiliations:** ^1^Department of Pharmacy, Obstetrics and Gynecology Hospital of Fudan University, Shanghai, China; ^2^Department of Pharmacology, School of Pharmacy, Fudan University, Shanghai, China; ^3^Department of Gastrointestinal Surgery, Harbin Medical University Cancer Hospital, Harbin, China; ^4^Department of Pharmacy, Huashan Hospital, Fudan University, Shanghai, China

**Keywords:** hydrogen sulfide, extravillous trophoblast, preeclampsia, apoptosis, oxidative stress

## Abstract

Extravillous trophoblast invasion disorder caused by oxidative stress is involved in the pathogenesis of preeclampsia (PE). In order to identify whether hydrogen sulfide (H_2_S) can prevent oxidative stress injury in extravillous trophoblasts. HTR-8/SVneo cells were detected by H_2_S inhibiting H_2_O_2_ induced oxidative mitochondrial damage. Reactive oxygen species (ROS) were detected, as well as malondialdehyde (MDA), catalase (CAT), and superoxide dismutase (SOD). JC-1 detected the potential of the mitochondrial membrane in this experiment. Then to detect the expression level of the apoptosis-inducing protein B-cell lymphoma-2 (Bcl-2) associated X protein (Bax), caspase 3, p53, p-p53, the apoptosis-inhibiting protein Bcl-2, PRAP, and the mitochondria fission protein Drp1, p-Drp1. CCK-8 assay, it was demonstrated that cell proliferation in the NaHS group was significantly higher than that in the Mod group, indicating that H_2_S may induce cell proliferation. Transwell assay elucidated that cell invasion in the NaHS group was recovered compared to the Mod group. ROS concentration no matter in cells or mitochondria was decreased by NaHS, which we could get from the comparison between the Mod group, PAG group, and NaHS group. The concentration of MDA was significantly lower in the NaHS group, and the concentration of SOD was extremely high in the NaHS group. Utilized JC-1 to detect mitochondrial membrane potential and found that cells from the NaHS group had a stable potential while cells from the Mod group and PAG group partly lost their potential, which could demonstrate that NaHS could maintain mitochondrial membrane potential. The western blot results revealed that p-Drp1 had a significant decline in the NaHS group, which means mitochondria fission was decreased in the NaHS group. The expression level of Bax and caspase 3 was significantly lower than in the Mod group and PAG group, and the expression level of Bcl-and PRAP was significantly higher in the NaHS group. That could prove that NaHS protect HTR-8/SVneo cell by inhibiting cell apoptosis. These promising results show that H_2_S elicits its effects on cell apoptosis by decreasing ROS concentration, maintaining mitochondrial membrane stability, and promoting apoptosis-inhibiting protein expression in cells.

## Introduction

There is no known pathophysiology for preeclampsia (PE), which is a pregnancy-related multisystem disorder. In developing countries, it accounts for 15% of all maternal deaths, neonatal and fetal deaths, and preterm births (1, 2). The condition is characterized by uncontrolled hypertension during pregnancy accompanied by proteinuria, with neurological symptoms such as seizures at the end of pregnancy (3, 4). Despite the fact that the pathophysiology of PE remains unclear, previous studies have indicated that it is closely associated with oxidative stress in trophoblast cells. This led to altered physiologic transformation of spiral arteries, leading to placental hypoperfusion and hypoxia, and ultimately placental insufficiency. During normal placental implantation, extravillous trophoblast migrates into the spiral arteries of the maternal uterus during placental implantation, forming vascular sinuses at the fetal-maternal interface to supply nutrients to the fetus (5). While during PE, trophoblast failed to remodel the spiral artery, leading to placental ischemia and other syndromes of PE.

Oxidative stress may occur as a result of hypoxia and reoxygenation of the placenta caused by a poor spiral artery invasion (6, 7). One of the key features of PE is oxidative stress in the trophoblast, which is partially caused by mitochondrial dysfunction (8). Mitochondrial outer membrane permeabilization (MOMP) is associated with mitochondrial function. When MOMP is destroyed, reactive oxygen species (ROS) increase and cell apoptosis are induced by mitochondrial pathways (9). Pores formed by Bax, a protein from the Bcl-2 family, contribute to the loss of MOMP and then release factors such as cytochrome c to activate caspase, which finally leads to cell apoptosis. Thus, mitochondria could be a trigger for curing PE.

H_2_S has been proved by many scientists that it may participate in a lot of pathophysiologic processes, which are quite important in the human body, such as apoptosis, oxidative stress, inflammation, and angiogenesis (10). As a physiologic vascular regulator, H_2_S inhibits proliferation and regulates apoptosis and autophagy of vascular cells, thus affecting vascular diseases in various ways (11). In the meantime, it is well known that H_2_S regulates long-term potentiation and calcium levels in neuronal cells, so disturbances of H_2_S levels in cells have been implicated in neurodegenerative diseases such as Alzheimer’s, Parkinson’s, strokes, and traumatic brain injuries, which attract a lot of attention (12). H_2_S has also been proven to promote cancer cell death, inhibit cancer angiogenesis, and metastasis (13). The mitochondrial enzyme superoxide dismutase (SOD) and ROS are regulated by H_2_S, which protects cardiomyocytes and inhibits apoptosis (14, 15). That is to say, H_2_S exerts its protective effect on cells through the mitochondria pathway. The L-cysteine/H_2_S pathway has been linked to the development of PE in previous studies (16), and H_2_S donors may have prevented PE (17–19). As we mentioned above, we postulate that H_2_S might be able to protect the placenta in PE. In line with our hypothesis, a study has demonstrated that AP39, a novel mitochondria-targeted hydrogen sulfide donor, could restore mitochondrial health through increasing active mitochondrial content, and decreasing superoxide production (20). We suppose to identify the protection of H_2_S on the trophoblast of PE and the mechanism behind that.

## Materials and methods

### Chemicals

NaHS was used as an H_2_S donor When NaHS reacts with water, it provides a solution of H_2_S with a concentration of approximately 33% of its original concentration (21). NaHS was purchased from Aladdin (China). Our previous experimental results showed that NaHS within 400 μM promoted the proliferation of HTR-8/Svneo cells. Referring to other kinds of literature and our result (22, 23), we chose the concentration of 200 μM for our experiments. PAG (DL-Propargylglycine), an endogenous hydrogen sulfide generating enzyme inhibitor was purchased from Sigma (USA). Dulbecco’s modified Eagle’s medium (DMEM), the cell culture medium, was purchased from BasalMedia (China), and fetal bovine serum (FBS), penicillin, and streptomycin were purchased from GIBCO-BRL (USA). CCK-8 (Cell Counting Kit-8) was purchased from Beyotime (China). Rabbit polyclonal antibodies to GAPDH, Bax, Bcl-2, p53, p-p53, pro-PRAP, and COX IV were purchased from Proteintech (USA). Mice antibodies to p-p53 and Drp1 were purchased from Bioss (USA), and antibodies to pro-caspase 3, and p-Drp1 were purchased from Cell Signaling Technology (USA).

### Cell culture

HTR-8/Svneo cell lines (HTR-8) were purchased from Shanghai Zhong Qiao Xin Zhou Biotechnology Co.,Ltd., DMEM supplemented with 10% fetal calf serum, 100 U/mL penicillin, and 100 g/mL streptomycin, at 37^°^C, 5% CO_2_, and saturated humidity were used to culture HTR-8 cells. A 0.25% concentration of trypsin was used to digest logarithmic phase cells for the experiment. A suspension of individual cells was prepared and seeded into an appropriate plate. A suitable condition for dividing cells was achieved by dividing them into four groups: the Control group, the Mod group, the PAG group and the NaHS group. After detection, we chose 0.1 mM as the final concentration of H_2_O_2_. Serum-free DMEM, H_2_O_2_ (0.1 mM), H_2_O_2_ (0.1 mM) and PAG (5 mM), H_2_O_2_ (0.1 mM), and NaHS (0.2 mM) were added into different groups at the same time for 24 h.

### CCK-8 assay

We evaluated cell viability by Cell counting kit 8 assay. Each group got 100 μL of one of the medicine solutions we mentioned above, and the Control group got 100 μL of serum-free DMEM medium. A 100 μL of CCK-8 was added to each culture plate hole after 24 h of cultivation and incubated at 37^°^C for 3 h. In order to determine the relative survival rate, we selected the wavelength of 450 nm and measured the absorbance of each well on the plate reader (Tecan, Germany, Infinite M Nano).

### Transwell assay

A concentration of 1 × 10^5^cells/well was seeded into the filters in 200 μL of FBS-free medium for 24 h, followed by a different treatment. In the lower chambers, 500 μL of medium containing 30% FBS was used. Using a tipped swab, cells on the top side were removed 24 h after treatment Crystal violet (E607309, Sangon Biotech, China) was used to stain cell migration to the lower side. For each group of testing, at least three independent wells were included, and five to six photographs were taken for each group, and the results were analyzed using Image J (Nation Institutes of Health, Bethesda, MD, USA).

### Assay of reactive oxygen species generation

A membrane-permeable fluorescent probe, 2′, 7′-dichlorofluorescin diacetate (DCFH-DA), and a fluorescence microscope (ECLIPSE NI, NIKON, Japan) were used to detect intracellular ROS. The fluorescence is detected at 525 nm after excitation at 488 nm. The concentration of mitochondrial ROS was detected utilizing MitoSOX Red Mitochondrial Superoxide Indicator (40778ES50, Yeasen, China) and fluorescence microscope (ECLIPSE NI, NIKON, Japan). The fluorescence is detected at 580 nm after excitation at 510 nm.

### Detection of mitochondrial membrane potential

An JC-1 array of fluorescent dyes were used to detect mitochondrial membrane potential (ΔΨ_m_) (10009172, Cayman, Japan). As mitochondrial membrane potential declines, the red fluorescence of the JC-1 assay changes to green. A total of 300 μL of JC-1 staining solution was added to each well after HTR-8 cells were treated with different medicine solutions for 24 h. A 20 min incubation period was performed in the incubator on the plate. Fluorescence microscopy was used to photograph the plate (ECLIPSE Ts2, NIKON, JapanJC-1 monomers were detected at 485 and 535 nm, and JC-1 aggregates were detected at 535 and 595 nm.

### Western blot analysis

First, cells were washed with PBS to remove any remaining DMEM on them, and then lysed on ice for 30 min in RIPA buffer (P0013C, Beyotime, China). The protein concentrations of all groups were determined using a BCA protein assay kit (DQ111-01, TransGen Biotech, China). Proteins (30 g) were separated on 10% polyacrylamide SDS gels for nearly 2 h, transferred onto PVDF membranes in running buffer, then blocked in 5% skim milk solution for 2 h following that, the membrane was incubated with polyclonal antibodies against GAPDH (60004-1-ig, Proteintech, America), Bax (50599-2-Ig, Proteintech, America), Bcl-2 (26593-1-AP, Proteintech, America), p53 (10442-1-AP, Proteintech, America), p-p53 (bs-3707R, Bioss, China), pro-PRAP (12926-1-AP, Proteintech, America), COX IV (66110-1-Ig, Proteintech, America), Drp1 (bs-4100R, Bioss, China), pro-caspase 3 (9662, Cell Signaling Technology, America), and p-Drp1 (3455, Cell Signaling Technology, America). As a secondary antibody, HRP-conjugated goat anti-rabbit IgG (A0208, Beyotime, China) and HRP-conjugated goat anti-mice IgG (A0216, Beyotime, China) were used on the second day for cleaning non-specific binding, then incubated for 1 h at room temperature. Chemiluminescence was performed using ECL ultra-sensitive light-emitting liquid (36208ES76, Yeasen, China) after the membranes had been washed with TBST buffer Chemiluminescence Imaging System (620028-08Q, CLINX, China) and Quantity One software (Bio-Rad, Hercules, CA, USA) were used to measure signal intensity.

### Analyses of statistics

The mean and standard deviation are expressed as mean ± SEM. For multiple comparisons, a one-way ANOVA was used followed by a *post hoc* analysis adjusted with a least-significant-difference correction (SPSS Inc., Chicago, IL, USA). The significance level was considered if *P < 0.05, ^**^P < 0.01, ^***^P < 0.001.

## Results

### H_2_O_2_ increased oxidative stress in HTR-8/SVneo cells

**FIGURE 1:**
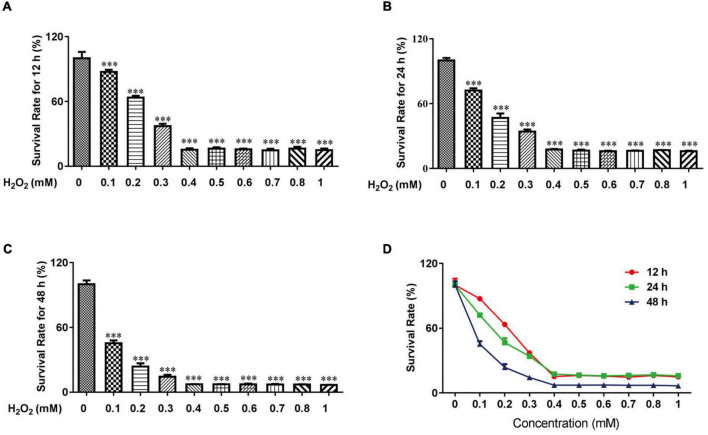
H_2_O_2_ caused oxidative stress damage in HTR-8/SVneo cells. The survival rate of HTR-8/SVneo cells after being treated with different doses of H_2_O_2_ for 12 h **(A)**, 24 h **(B)** and 48 h **(C)**. Summary of HTR-8/SVneo cells survival rate after 12, 24, and 48 h **(D)**. The data are expressed as mean ± SEM. ^***^Vs. Control, *P* < 0.001.

Previous studies demonstrated that H_2_O_2_ could be used to increase oxidative stress in HTR-8 cells (24, 25). For experiments with CCK-8 cells, 0.1 mM H_2_O_2_ for 12 h induced mild apoptosis in HTR-8 cells, moderate apoptosis at 24 h, and severe apoptosis at 48 h (Figure 1). We finally chose 0.1 mM H_2_O_2_ treated for 24 h to continue our following experiments.

### H_2_O_2_-induced cell apoptosis

**FIGURE 2:**
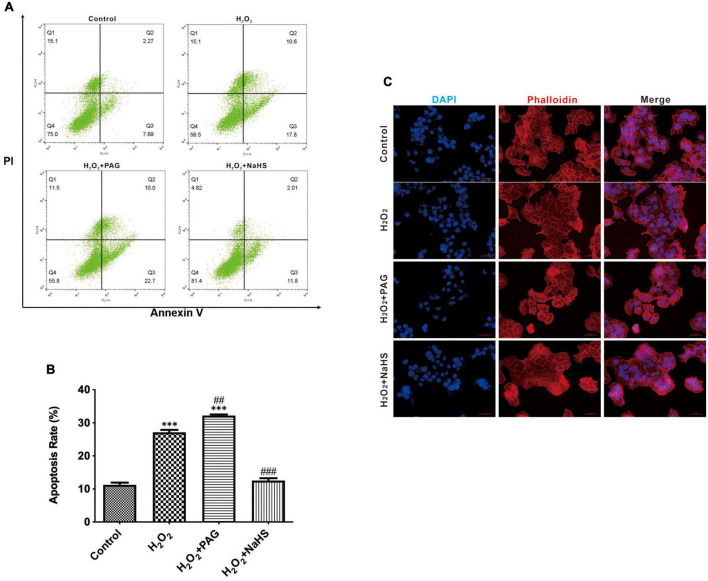
H_2_O_2_ induced HTR-8/SVneo cells apoptosis. The proportion of apoptotic cells was detected by Annexin V. **(A)** Representative pictures of Annexin IV and PI-derived fluorescence in HTR-8/SVneo cells after different treatments. **(B)** Summarize the flow cytometry results. **(C)** Different groups of cells had different morphology changes in the cell nucleus (400×). The data are expressed as mean ± SEM. ^***^Vs. Control, *P* < 0.001. ^##^*Vs.* H_2_O_2_, *P* < 0.01. ^###^*Vs.* H_2_O_2_, *P* < 0.001. Bar = 50 μm.

We utilized DAPI and flow cytometry to confirm cell apoptosis. After attaching to AT regions of DNA, DAPI stains nuclear and chromosomes, which emit blue fluorescence. Thus, it can indicate morphological changes in the nuclear. Nuclear sequestration is a distinctive feature of apoptosis, and DAPI stains the nucleus and shows its morphology. While apoptosis occurs, Phosphatidylserine (PS) transfers from the cell’s inner membrane to the outer membrane. Thus, we could use Annexin V to combine PS in the outer membrane of cells to indicate apoptotic cells. PAG is one of the cystathionine γ-lyase (CSE) inhibitors, which can inhibit the endogenous synthesis of H_2_S. As shown in Figure 2C, after being treated with H_2_O_2_, we can easily find the change in nuclear morphology from round shapes to irregular shapes. Treated with H_2_O_2_ and PAG even increased the sequestration, indicating that H_2_S might be involved in the HTR-8 cell apoptosis process. Meanwhile, the result of the NaHS (0.2 mM) group showed that NaHS can partly relieve the influence of H_2_O_2_. Flow cytometry results gave further proof of the above results. Figures 2A,B presented that the proportion of apoptotic cells in the H_2_O_2_ group and the PAG group were extremely higher than in the control group and the NaHS group (*P* < 0.05). The results demonstrated that H_2_O_2_ induces HTR-8 cell apoptosis and NaHS could protect HTR-8 cells against H_2_O_2_-induced injury.

### NaHS promoted cell proliferation and invasion after H_2_O_2_ treatment

**FIGURE 3:**
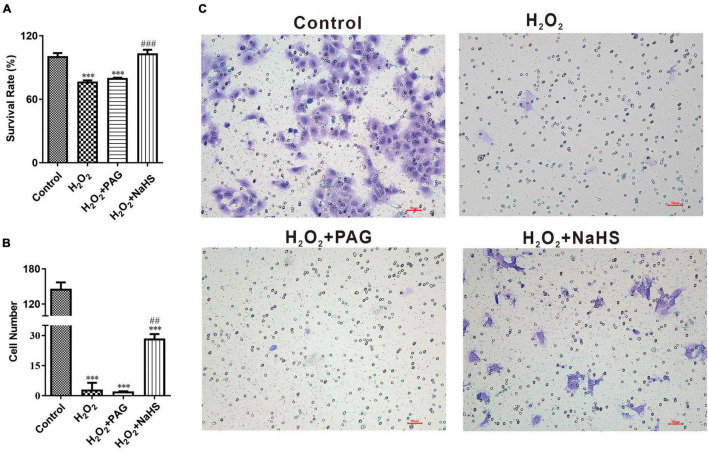
NaHS induced HTR-8/SVneo cells invasion and proliferation. **(A)** Summarize of survival rate of HTR-8/SVneo cells after different treatments. **(B)** Summarize cell number detected by invasion assay. **(C)** Representative picture of HTR-8/SVneo cells stained with crystal violet. The data are expressed as mean ± SEM. ^***^Vs. Control, *P* < 0.001. ^##^*Vs.* H_2_O_2_, *P* < 0.01. ^###^*Vs.* H_2_O_2_, *P* < 0.001.

In order to demonstrate the effect of NaHS, we analyzed cell proliferation ability by CCK-8 assay and cell invasion ability by invasion assay. CCK-8 assay represented that the survival rate of the NaHS group was significantly higher than the Mod group and the PAG group, indicating that H_2_S could induce HTR-8 cells proliferation at 0.2 mM after H_2_O_2_ treatment, as shown in Figure 3A. The invasion assay showed that there were more HTR-8 cells transferring to the lower chambers in the NaHS group (Figures 3B,C), by which we could demonstrate that H_2_S had the ability to accelerate HTR-8 cell invasion. While in the Mod group, cells were treated with 0.1 mM H_2_O_2_ for 24 h, thus causing the attenuation of cell invasion. Above all, we could find that H_2_S was sufficient to protect HTR-8 cells from oxidative stress, because of its ability to induce cell proliferation and cell invasion.

### NaHS reduced the concentration of reactive oxygen species

**FIGURE 4:**
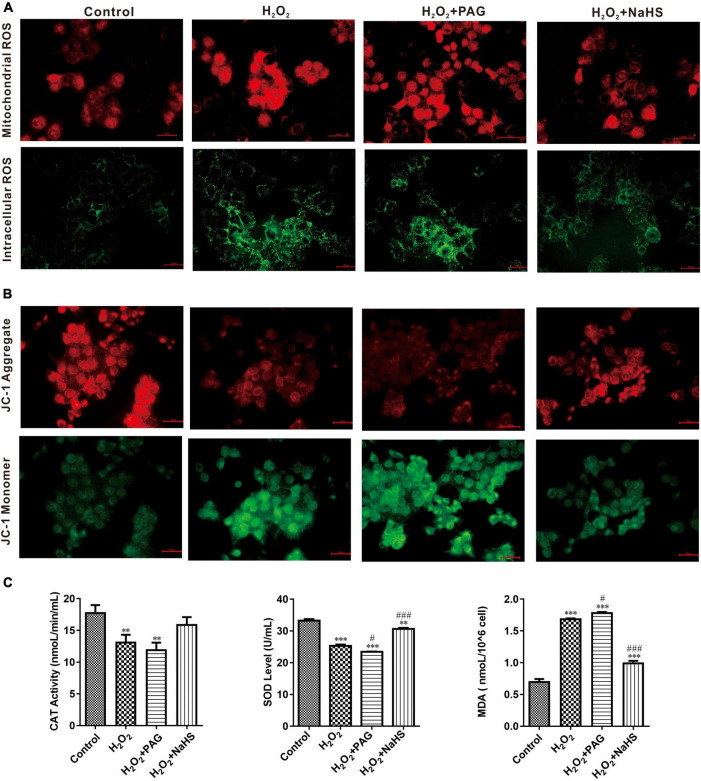
NaHS inhibited oxidative stress in HTR-8/SVneo cells. **(A)** The images of different group were obtained with excitation/emission of 525/590 nm for red fluorescence and 490/530 nm for green fluorescence **(B)**. NaHS decreased mitochondrial membrane potential (ΔΨ_m_) in HTR-8/SVneo cells. Red fluorescence and green fluorescence intensity for JC-1 staining of different groups. Bar = 50 μm. **(C)** Effect of NaHS on CAT, SOD and MDA. The data are expressed as mean ± SEM. ^**^Vs. Control, *P* < 0.01. ^***^Vs. Control, *P* < 0.001. ^#^*Vs.* H_2_O_2_, *P* < 0.05. ^###^*Vs.* H_2_O_2_, *P* < 0.001.

A ROS is a molecule that contains oxygen and reacts chemically. Excessive ROS can induce apoptosis through both the different pathways and thus do harm to the human body (26). We detected the concentration of intracellular and mitochondrial ROS utilizing DCFH-DA (for cell) and MitoSOX Red (for mitochondria). DCFH-DA is a cell-permeable probe that could be oxidized by ROS and emits green fluorescence, and MitoSOX Red could penetrate into cells and selectively targets mitochondria to visualize the process of ROS production in the mitochondria. In Figure 4A, red fluorescence intensity is significantly higher after H_2_O_2_ treatment, especially in H_2_O_2_ plus PAG group. And the intensity suppressed in the NaHS group demonstrated that H_2_S could inhibit the production of mitochondrial ROS. It is also obvious that NaHS decreased ROS levels in cells. In line with a previous study, H_2_S exerts its antioxidant effect by decreasing ROS.

SOD and catalase (CAT) have an important role in scavenging the levels of ROS. Their activity and concentration are highly related to the oxidative condition in cells. Malondialdehyde (MDA) is cytotoxic and commonly used as a lipid peroxidation indicator. After detecting the concentration of SOD, CAT, and MDA (in Figure 4C), we could find a higher level of MDA and lower levels of SOD and CAT in the H_2_O_2_ group and the PAG group, which is consistent with the previous results of ROS. After being treated with NaHS, the concentration of SOD and CAT became significantly higher which indicated that H_2_S could protect HTR-8 cells through cleaving ROS.

### NaHS restored mitochondrial function

Perturbation of mitochondrial function is associated with loss of the mitochondrial transmembrane potential and the release of apoptogenic factors. Mitochondrial membrane potential (ΔΨm) is a sensitive indicator of mitochondrial damage (27). JC-1 is a commonly used fluorescent probe, which is used for the detection of ΔΨ_m_. When ΔΨ_m_ is high, JC-1 emits red fluorescence. Unlike in a high ΔΨm condition, it is not possible for JC-1 to gather together at lower ΔΨmin the mitochondrial matrix and produce green fluorescence. As shown in Figure 4B, H_2_O_2_ treatment for 24 h could extremely increase green fluorescence, and H_2_O_2_ plus PAG undoubtedly made the green fluorescence lighter, which means H_2_O_2_ has a harmful effect on mitochondrial membrane potential. NaHS reduced the effects of H_2_O_2_ on ΔΨ_m_ which is proved by the increased red fluorescence in the NaHS group.

**FIGURE 5:**
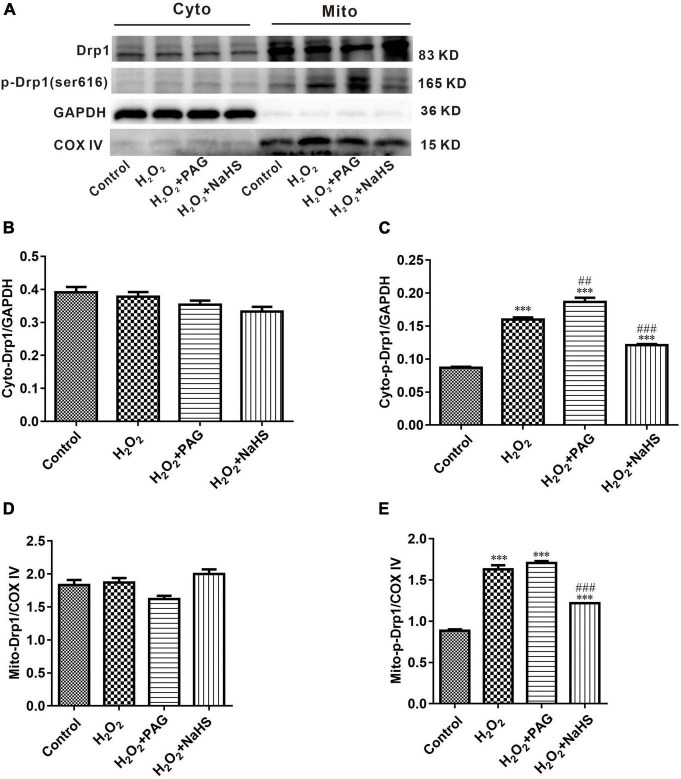
Effect of NaHS on mitochondrial cleavage and fusion proteins activity in HTR-8/SVneo cells. **(A)** Representative Western blot showing Drp1, p-Drp1 in cytoplasm and mitochondria in HTR-8/SVneo cells after different treatments. **(B–E)** Bar charts indicating different proteins between different groups. Results in the cytoplasm were normalized against GAPDH, and results in mitochondria were normalized against COX IV. The data are expressed as mean ± SEM. ^***^Vs. Control, *P* < 0.001. ^##^*Vs.* H_2_O_2_, *P* < 0.01. ^###^*Vs.* H_2_O_2_, *P* < 0.001.

Mitochondria are complex organelles, and their cleavage and fusion are precisely regulated by genes, but this balance can be disrupted when apoptosis occurs. In the pro-apoptotic phase, mitochondria undergo cleavage and release a series of pre-apoptotic signals (28). Many factors control mitochondrial cleavage and fusion. One such factor is the family of kinetic-related GTPases (Drp1). Drp1 can promote mitochondrial cleavage when phosphorylated at serine 616 (ser616) (29). Western blot was used to detect the expression of p-Drp1 and Drp1 within and outside the mitochondria. As shown in Figure 5, compared with the Control group, the H_2_O_2_ group and the PAG group showed a significant increase in p-Drp1 inside and outside the mitochondria. The NaHS group showed a significant and extreme decrease in p-Drp1 compared with the H_2_O_2_ group and the PAG groups. This suggests that H_2_O_2_ and PAG can promote mitochondrial lysis, implying that this lysis may be related to H_2_S concentration. And NaHS have a protective effect on mitochondria by inhibiting its lysis, from which we purposed that it might be related to cell apoptosis.

### NaHS protected HTR-8/SVneo cells by inhibiting cell apoptosis

**FIGURE 6:**
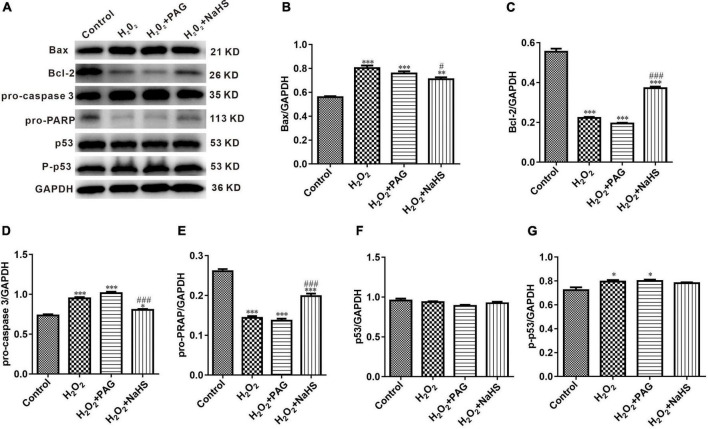
Effect of NaHS on cell apoptosis pathway proteins activity in HTR-8/SVneo cells. **(A)** Representative western blot showing Bax, Bcl-2, pro-caspase 3, pro-PRAP, p53, and p-p53 in HTR-8/SVneo cells after different treatments. **(B–G)** Bar charts indicating different apoptosis-related proteins between different groups. Results were normalized against GAPDH. The data are expressed as mean ± SEM. *Vs. Control, *P* < 0.05. ^***^Vs. Control, *P* < 0.001. ^#^*Vs.* H_2_O_2_, *P* < 0.05. ^###^*Vs.* H_2_O_2_, *P* < 0.001.

Western Blot results indicated the condition of cell apoptosis more clearly. As shown in Figure 6, apoptosis-promoting protein Bax, caspase-3 and phosphorylated-p53 demonstrated an increasing trend in the H_2_O_2_ group and the PAG group, while apoptosis-inhibiting protein Bcl-2 and PRAP demonstrated a reversed trend. The condition is totally different in the NaHS group. The concentration of Bcl-2 and PRAP was significantly higher than in the H_2_O_2_ group and the PAG group, and caspase 3 was significantly lower in the NaHS group. The results showed the induce-apoptosis effect of H_2_O_2_ and the protective effect of H_2_S on HTR-8 cells at the protein level through the cell apoptosis pathway.

## Discussion

There are many complications associated with pregnancy, but one of the most feared is preeclampsia. PE usually begins as hypertension and proteinuria in the third trimester and can quickly progress to more serious conditions, such as death for both mother and child. It is still unclear what causes PE, but clinical and pathological studies suggest the placenta plays a pivotal role in their pathogenesis (5). Also, endogenous H_2_S pathway could contribute to the development of PE both in gene level and protein level (30). Based on previous studies, abnormal placental development and angiogenesis are key features of PE, which progresses and eventually leads to ischemia and hypoxia of the placenta. The excessive apoptosis of trophoblast cells is one of the factors that subsequently lead to poor placental development and angiogenesis. In this study, we verified the inhibitory effect of H_2_S on apoptosis of trophoblast cells and further investigated its mechanism. Finally, we revealed the protective effect of H_2_S on the placenta in PE and its mechanism.

HTR-8/Svneo cell line is an immortalized extravillous trophoblast cell line derived from normal human early pregnancy placenta. It is a common cell line used to study the function of early pregnancy trophoblast *in vitro* (31). In this experiment, we confirmed that H_2_S could promote the proliferation and invasion of HTR-8 cells after H_2_O_2_ treatment which is consistent with our previous study (23), and its antioxidant effect reduced the production of ROS, maintained the function of mitochondria, and suppress the synthesis of apoptosis-related proteins. To confirm the role of H_2_S in promoting cell proliferation and invasion, CCK-8 assay and invasion assay were utilized to detect both the proliferation and invasion abilities of cells, and the results showed that the survival rate of cells and the migration of cells were significantly increased after the addition of NaHS compared with the Mod group. This verified the protective effect of H_2_S on HTR-8 cells under excessive oxidative stress.

H_2_S inhibits cell apoptosis at low concentrations. There are three ways in which H_2_S inhibits apoptosis: First, it regulates the MAPK pathway through ATP-sensitive potassium channels and thus inhibits apoptosis; Second, H_2_S is oxidized in the cytoplasm and can hypersulfide caspase 9 and other proteins, thus disabling them and the subsequent apoptotic process will be inhibited; Third, H_2_S hypersulfates NF-κB and directly induced it’s combining with anti-apoptosis gene promoter. H_2_S exerts its effect on mitochondria through regulating mitochondrial fission and fusion, which is achieved by regulating the Drp1 protein, a major factor controlling mitochondrial fission, which can form helical oligomers and thus contribute to mitochondrial division (32). Our study confirmed that low concentrations of H_2_S can reduce the intracellular p-Drp1/Drp1 ratio, thus reducing mitochondrial cleavage and further reducing the accumulation of ROS and thus inhibiting apoptosis. We also demonstrated that H_2_S could restore mitochondrial membrane potential, reduce mitochondrial cleavage, and decrease ROS levels in mitochondria, which lead to the inference that the inhibitory effect of H_2_S on apoptosis in HTR-8 cells is partly dependent on the mitochondrial pathway.

p53 is an intracellular antitumor factor that is used in the field of tumor therapy because of its ability to inhibit cell growth and promote apoptosis. When cells are stimulated by an internal or external environment (e.g., DNA damage or hypoxia), the p53 expression level will be up-regulated, thus causing apoptosis. p53 expression promotes the up-regulation of the expression of the pro-apoptotic signal Bax (Bcl-2 associated × protein) and the down-regulation of the expression of the anti-apoptotic signal Bcl-2 in the Bcl-2 gene family. The Bax protein forms pores in the mitochondrial membrane, resulting in destroying mitochondrial permeability and subsequent loss of mitochondrial membrane potential and release of cytochrome c (33). Caspase 9, a protein of the aspartate family (caspase family) that is essential in the mitochondrial apoptotic pathway, is upstream of the entire cascade reaction and can have an effect by activating downstream acting proteins, and is an initiator protein. When activated, the initiator protein can activate downstream caspase 3, thus activating DNA degradation by deoxyribonucleases, triggering apoptosis. Our study confirmed that H_2_S can inhibit apoptosis in the mitochondrial pathway by down-regulating the expression of Bax and caspase 3 proteins and up-regulating the expression of Bcl-2, PRAP proteins.

However, many details still need to be added in the future. We verified at the cellular level that H_2_S inhibited apoptosis of HTR-8 cells after H_2_O_2_ treatment *via* the mitochondrial pathway, and confirmed that its antioxidant effect was sufficient to promote the proliferation and invasion of HTR-8 cells after H_2_O_2_ treatment. PE is a disease which is quite difficult to research, because of its unique characteristics, we here provide a potential way to cure or prevent the happening of PE. And we also replenish the function of H_2_S which is a core-compound in our laboratory. Even though, these results need to be confirmed at the animal level in order to have a better clinical meaning.

## Data availability statement

The original contributions presented in the study are included in the article/supplementary material, further inquiries can be directed to the corresponding author/s.

## Author contributions

WG and XW designed the study. XW, SY, and JW wrote the manuscript, originated the central idea, and supervised the work. XW, SY, YJ, HP, JG, and WG performed experiments and analyzed the data. HP and JW contributed the experiment materials supply. XW, SY, YJ, HP, JG, JW, and WG analyzed the data and read the manuscript. All authors contributed to the article and approved the submitted version.

## References

[1] Nirupama R Divyashree S Janhavi P Muthukumar SP Ravindra PV. Preeclampsia: pathophysiology and management. *J Gynecol Obstet Hum Reprod.* (2021) 50:101975. 10.1016/j.jogoh.2020.101975 33171282

[2] Committee on Practice Bulletins–Obstetrics. Gestational hypertension and preeclampsia: ACOG practice bulletin summary, number 222. *Obstet Gynecol.* (2020) 135:1492–5. 10.1097/AOG.0000000000003892 32443077

[3] Raguema N Moustadraf S Bertagnolli M. Immune and apoptosis mechanisms regulating placental development and vascularization in preeclampsia. *Front Physiol.* (2020) 11:98. 10.3389/fphys.2020.00098 32116801 PMC7026478

[4] Erez O Romero R Jung E Chaemsaithong P Bosco M Suksai M Preeclampsia and eclampsia: the conceptual evolution of a syndrome. *Am J Obstet Gynecol.* (2022) 226:S786–803. 10.1016/j.ajog.2021.12.001 35177220 PMC8941666

[5] Rana S Lemoine E Granger JP Karumanchi SA. Preeclampsia: pathophysiology, challenges, and perspectives. *Circ Res.* (2019) 124:1094–112. 10.1161/CIRCRESAHA.118.313276 30920918

[6] Chiarello DI Abad C Rojas D Toledo F Vázquez CM Mate A Oxidative stress: normal pregnancy versus preeclampsia. *Biochim Biophys Acta Mol Basis Dis.* (2020) 1866:165354. 10.1016/j.bbadis.2018.12.005 30590104

[7] Taysi S Tascan AS Ugur MG Demir M. Radicals, oxidative/nitrosative stress and preeclampsia. *Mini Rev Med Chem.* (2019) 19:178–93. 10.2174/1389557518666181015151350 30324879

[8] Zsengellér ZK Rajakumar A Hunter JT Salahuddin S Rana S Stillman IE Trophoblast mitochondrial function is impaired in preeclampsia and correlates negatively with the expression of soluble fms-like tyrosine kinase 1. *Pregnancy Hypertens.* (2016) 6:313–9. 10.1016/j.preghy.2016.06.004 27939475

[9] Dadsena S King LE García-Sáez AJ. Apoptosis regulation at the mitochondria membrane level. *Biochim Biophys Acta Biomembr.* (2021) 1863:183716. 10.1016/j.bbamem.2021.183716 34343535

[10] Shen Y Shen Z Luo S Guo W Zhu YZ. The cardioprotective effects of hydrogen sulfide in heart diseases: from molecular mechanisms to therapeutic potential. *Oxid Med Cell Longev.* (2015) 2015:925167. 10.1155/2015/925167 26078822 PMC4442295

[11] Lv B Chen S Tang C Jin H Du J Huang Y. Hydrogen sulfide and vascular regulation – an update. *J Adv Res.* (2021) 27:85–97. 10.1016/j.jare.2020.05.007 33318869 PMC7728588

[12] Kumar M Sandhir R. Hydrogen sulfide in physiological and pathological mechanisms in brain. *CNS Neurol Disord Drug Targets.* (2018) 17:654–70. 10.2174/1871527317666180605072018 29866024

[13] Lee ZW Deng LW. Role of H2S donors in cancer biology. *Handb Exp Pharmacol.* (2015) 230:243–65. 10.1007/978-3-319-18144-8_13 26162839

[14] Murphy B Bhattacharya R Mukherjee P. Hydrogen sulfide signaling in mitochondria and disease. *Faseb J.* (2019) 33:13098–125. 10.1096/fj.201901304R 31648556 PMC6894098

[15] Guo W Kan JT Cheng ZY Chen JF Shen YQ Xu J Hydrogen sulfide as an endogenous modulator in mitochondria and mitochondria dysfunction. *Oxid Med Cell Longev.* (2012) 2012:878052. 10.1155/2012/878052 23304257 PMC3523162

[16] Sarno L Raso GM di Villa Bianca RD Mitidieri E Maruotti GM Esposito G OS064. Contribute of the L-cysteine/H2S pathway in placenta homeostasisin hypertensive disorders. *Pregnancy Hypertens.* (2012) 2:211–2. 10.1016/j.preghy.2012.04.065 26105277

[17] Rezai H Ahmad S Alzahrani FA Sanchez-Aranguren L Dias IH Agrawal S MZe786, a hydrogen sulfide-releasing aspirin prevents preeclampsia in heme oxygenase-1 haplodeficient pregnancy under high soluble flt-1 environment. *Redox Biol.* (2021) 38:101768. 10.1016/j.redox.2020.101768 33137710 PMC7610044

[18] Saif J Ahmad S Rezai H Litvinova K Sparatore A Alzahrani FA Hydrogen sulfide releasing molecule MZe786 inhibits soluble Flt-1 and prevents preeclampsia in a refined RUPP mouse model. *Redox Biol.* (2021) 38:101814. 10.1016/j.redox.2020.101814 33321463 PMC7744945

[19] Terstappen F Clarke SM Joles JA Ross CA Garrett MR Minnion M Sodium thiosulfate in the pregnant dahl salt-sensitive rat, a model of preeclampsia. *Biomolecules.* (2020) 10:302. 10.3390/biom10020302 32075042 PMC7072460

[20] Covarrubias AE Lecarpentier E Lo A Salahuddin S Gray KJ Karumanchi SA AP39, a modulator of mitochondrial bioenergetics, reduces antiangiogenic response and oxidative stress in hypoxia-exposed trophoblasts: relevance for preeclampsia pathogenesis. *Am J Pathol.* (2019) 189:104–14. 10.1016/j.ajpath.2018.09.007 30315766 PMC6854435

[21] Shen Y Guo W Wang Z Zhang Y Zhong L Zhu Y. Protective effects of hydrogen sulfide in hypoxic human umbilical vein endothelial cells: a possible mitochondria-dependent pathway. *Int J Mol Sci.* (2013) 14:13093–108. 10.3390/ijms140713093 23799362 PMC3742176

[22] Xin L Junhua W Long L Jun Y Yang X. Exogenous hydrogen sulfide protects SH-SY5Y cells from OGD/Rinduced injury. *Curr Mol Med.* (2017) 17:563–7. 10.2174/1566524018666180222121643 29473502

[23] Wang XL Tang J. Focal adhesion kinase signaling is necessary for the hydrogen sulfide-enhanced proliferation, migration, and invasion of HTR8/SVneo human trophoblasts. *Reprod Dev Med.* (2022). 10.1097/RD9.0000000000000047 [Epub ahead of print].

[24] Qi L Jiang J Zhang J Zhang L Wang T. Curcumin protects human trophoblast HTR8/SVneo cells from H(2)O(2)-induced oxidative stress by activating Nrf2 signaling pathway. *Antioxidants (Basel).* (2020) 9:121. 10.3390/antiox9020121 32024207 PMC7071057

[25] Fu J-Y Jing Y Xiao Y-P Wang X-H Guo Y-W Zhu Y-J. Astaxanthin inhibiting oxidative stress damage of placental trophoblast cells in vitro. *Syst Biol Reprod Med.* (2021) 67:79–88. 10.1080/19396368.2020.1824031 33103484

[26] Zhang J Wang X Vikash V Ye Q Wu D Liu Y ROS and ROS-mediated cellular signaling. *Oxid Med Cell Longev.* (2016) 2016:4350965. 10.1155/2016/4350965 26998193 PMC4779832

[27] Zorova LD Popkov VA Plotnikov EY Silachev DN Pevzner IB Jankauskas SS Mitochondrial membrane potential. *Anal Biochem.* (2018) 552:50–9. 10.1016/j.ab.2017.07.009 28711444 PMC5792320

[28] Cereghetti GM Stangherlin A Martins de Brito O Chang CR Blackstone C Bernardi P Dephosphorylation by calcineurin regulates translocation of Drp1 to mitochondria. *Proc Natl Acad Sci USA.* (2008) 105:15803–8. 10.1073/pnas.0808249105 18838687 PMC2572940

[29] Cribbs JT Strack S. Reversible phosphorylation of Drp1 by cyclic AMP-dependent protein kinase and calcineurin regulates mitochondrial fission and cell death. *EMBO Rep.* (2007) 8:939–44. 10.1038/sj.embor.7401062 17721437 PMC2002551

[30] Holwerda K Weedon-Fekjær S Staff A Nolte I Goor H Lely T PP013. Single nucleotide polymorphisms of the maternal cystathionine-b-synthase gene are associated with preeclampsia (PE). *Pregnancy Hypertens.* (2013) 3:72. 10.1016/j.preghy.2013.04.041 26105871

[31] Abbas Y Turco MY Burton GJ Moffett A. Investigation of human trophoblast invasion in vitro. *Hum Reprod Update.* (2020) 26:501–13. 10.1093/humupd/dmaa017 32441309 PMC7473396

[32] Fonseca TB Sánchez-Guerrero Á Milosevic I Raimundo N. Mitochondrial fission requires DRP1 but not dynamins. *Nature.* (2019) 570:E34–42. 10.1038/s41586-019-1296-y 31217603

[33] Shen Y White E. p53-dependent apoptosis pathways. *Adv Cancer Res.* (2001) 82:55–84. 10.1016/S0065-230X(01)82002-9 11447765

